# Exploring the Influence of Napping Habits on Job Satisfaction: A Quasi-Natural Experimental Study Based on Longitudinal Data from China

**DOI:** 10.3390/bs15060770

**Published:** 2025-06-03

**Authors:** Xin Liu, Xiaochong Wei, Longxin Zhang

**Affiliations:** School of Public Administration and Policy, Renmin University of China, Beijing 100872, China; lxin@ruc.edu.cn (X.L.); wyc36@ruc.edu.cn (X.W.)

**Keywords:** nap, nap duration, job satisfaction, Non-work and Sleep Framework (WNSF), quasi-natural experiment

## Abstract

Sleep behaviors, particularly midday rest periods, represent an often overlooked yet potentially significant factor in workplace attitudes and experiences. Despite their common practice in many cultures, the relationship between these restorative breaks and employees’ evaluations of their work environment remains underexplored in organizational research. This study investigates this relationship using longitudinal data from the China Family Panel Studies (CFPS), applying the work, non-work, and sleep framework (WNSF) as our theoretical foundation. Through a quasi-natural experimental approach, we discovered that midday rest periods positively influence workplace evaluations, but in a nuanced manner. Our findings reveal a pronounced inverted U-shaped relationship, suggesting that both insufficient and excessive duration of these breaks might diminish their benefits, while optimal duration maximizes positive outcomes. We strengthened these conclusions through rigorous methodological approaches including instrumental variable techniques, sensitivity analysis, treatment effect models, and matching models. The consistency of results across multiple analytical approaches corroborates our findings. This research both validates and extends the WNSF, highlighting the importance of strategic rest periods in organizational settings and offering practical insights for workplace policy development that can benefit both individuals and organizations in contemporary work environments.

## 1. Introduction

Sleep behaviors represent a fundamental yet underexplored determinant of workplace attitudes and organizational outcomes. While extensive research has examined traditional antecedents of job satisfaction—including demographic characteristics, job design, and organizational factors (Kooij et al., 2010; Aydin et al., 2012; Judge et al., 2002), the role of strategic napping in enhancing employee wellbeing and work attitudes remains poorly understood. This gap is particularly significant given that job satisfaction, defined as an employee’s positive emotional state resulting from job appraisal, constitutes one of the most critical predictors of performance, turnover, and organizational effectiveness (Weiss, 2002; X. Liu et al., 2025).

The emerging literature on sleep and workplace outcomes reveals three critical limitations that this study addresses. First, existing research predominantly employs cross-sectional designs with limited samples, constraining causal inferences about the napping–satisfaction relationship (De Bloom et al., 2017). Second, most studies focus on specific organizational contexts, particularly healthcare settings, limiting generalizability to broader work environments (Deng et al., 2024; Steven & Redfern, 2024). Third, and most importantly, previous research has largely treated napping as a binary phenomenon or assumed linear effects, overlooking the potentially crucial non-linear relationship between nap duration and workplace outcomes (Leng & Yaffe, 2019).

This study adopts the work, non-work, and sleep framework (WNSF) as its theoretical foundation (Crain et al., 2018). The WNSF conceptualizes sleep as a critical resource that influences work attitudes through energy restoration and cognitive enhancement mechanisms, while recognizing time as a finite resource requiring strategic allocation between work and recovery activities. However, the framework lacks a specification of the optimal sleep parameters and boundary conditions under which sleep behaviors maximize workplace benefits.

We theoretically propose and empirically test an inverted U-shaped relationship between nap duration and job satisfaction, grounded in three interconnected mechanisms. Physiologically, short naps (10–30 min) promote alertness and cognitive restoration without inducing sleep inertia, whereas longer naps trigger deeper sleep stages that result in temporary post-awakening impairment (Brooks & Lack, 2006). Resource-theoretically, brief naps represent efficient recovery strategies that minimize time away from work activities, while excessive napping creates time conflicts and stress that counteract recovery benefits (Crain et al., 2018). Psychologically, napping benefits follow diminishing returns patterns, with medium-length naps showing optimal effects on mood regulation and cognitive performance (Yang et al., 2024).

This research makes four significant theoretical and practical contributions. Methodologically, we employ a quasi-natural experimental approach with longitudinal Chinese data and instrumental variable techniques to establish causal relationships between napping behaviors and job satisfaction, addressing critical limitations of previous correlational studies. Theoretically, we provide the first systematic empirical evidence for the curvilinear effect of nap duration on job satisfaction, identifying optimal nap zones that extend and refine the WNSF framework. Contextually, we demonstrate how sleep behaviors operate within cultural contexts where napping is institutionally normalized, offering insights for global organizational policy development. Practically, our rigorous analytical approach, incorporating sensitivity analyses and matching models, provides evidence-based foundations for workplace napping policies that can simultaneously enhance employee well-being and organizational outcomes.

## 2. Napping Behavior and Job Satisfaction

The work, non-work, and sleep framework (WNSF) provides the theoretical foundation for understanding how napping behaviors influence job satisfaction through systematic resource allocation and energy management processes (Crain et al., 2018). The WNSF conceptualizes human energy as comprising physical energy (capacity to perform work) and energetic activation (vitality and enthusiasm), both of which are directly affected by sleep behaviors. Critically, the framework positions time as a finite resource requiring strategic allocation between work activities and recovery processes, establishing the theoretical basis for understanding why napping duration, rather than mere occurrence, determines workplace outcomes. This theoretical perspective suggests that the relationship between napping and job satisfaction operates through complex, interconnected mechanisms that collectively explain both the positive effects of strategic napping and the potential negative consequences of suboptimal nap durations.

The relationship between napping and job satisfaction operates primarily through three interconnected theoretical mechanisms that form an integrated framework for understanding workplace sleep behaviors. From a resource recovery perspective, napping represents a strategic intervention for replenishing depleted cognitive and emotional resources that accumulate during continuous work demands (Dutheil et al., 2021). As employees experience resource depletion manifested as increased fatigue, decreased concentration, and reduced emotional regulation capacity—all directly affecting job satisfaction—napping activates parasympathetic nervous system responses, reducing stress hormone levels while promoting beneficial neurotransmitter activity (Faraut et al., 2015; Milner & Cote, 2009). Empirical evidence consistently demonstrates that brief naps of 10–20 min significantly reduce both subjective fatigue and objective stress indicators, enabling employees to evaluate their work environment more positively and restore cognitive resource reserves (Brooks & Lack, 2006). This resource recovery process directly alleviates the negative effects of work stress, creating a pathway through which strategic napping enhances job satisfaction by enabling more positive workplace evaluations.

Simultaneously, napping significantly enhances multiple cognitive domains critical for job performance and satisfaction, including working memory capacity, information processing speed, decision-making quality, and creative thinking ability (Lovato & Lack, 2010). These cognitive improvements enable employees to meet work challenges more effectively, reduce errors, and achieve higher performance standards, generating enhanced feelings of competence and achievement that directly translate into job satisfaction. According to cognitive appraisal theory, when employees perceive themselves as capable of meeting work demands through enhanced cognitive functioning, job satisfaction naturally increases. Neuroimaging studies reveal that strategic napping enhances prefrontal cortex activity and executive function without disrupting circadian rhythms, providing biological evidence for the cognitive enhancement pathway (Wong et al., 2013). The third mechanism involves emotional regulation, where napping profoundly influences emotional well-being through neuroendocrine system modulation, particularly rebalancing limbic system and prefrontal cortex interactions (Goldschmied et al., 2015). Research demonstrates substantial improvements in emotional states following napping, with negative affect reductions of 30–40% and positive affect improvements of 20–35% (Taub & Berger, 1976; Kaida et al., 2006). According to affective events theory, these enhanced emotional experiences accumulate and directly influence work attitudes, creating a direct pathway from emotional regulation to job satisfaction.

While napping provides substantial benefits through these mechanisms, the WNSF’s emphasis on time as a finite resource suggests that these benefits follow a curvilinear rather than linear pattern, supported by optimization theory and resource balance models. This theoretical prediction indicates that the relationship between nap duration and job satisfaction reflects a critical transition between increasing and diminishing returns phases. Within the optimal duration zone of 10–30 min, napping occurs primarily in light sleep stages, providing mental restoration with minimal sleep inertia and maximizing positive effects on work performance and satisfaction (Brooks & Lack, 2006). This “efficient recovery interval” represents the optimal balance between resource restoration and time investment, where physiological and psychological benefits are proportional to the time allocated. However, beyond this optimal duration threshold, extended napping triggers multiple inhibitory factors that progressively diminish benefits. Sleep physiology research indicates that naps exceeding 30 min significantly increase deep sleep probability, resulting in post-awakening sleep inertia characterized by impaired cognitive and psychomotor function lasting 30–60 min (Hilditch & McHill, 2019). Additionally, organizational time resource theory suggests that extended napping creates time conflicts and task accumulation pressures that offset recovery benefits, while social cognitive factors introduce perceived risks of negative peer and supervisor evaluations (Mullins et al., 2014; Takahashi et al., 2004).

Converging evidence from multiple research domains supports this theoretical framework and demonstrates the complexity of napping effects on workplace outcomes. Studies consistently demonstrate that brief naps enhance alertness, mood, and cognitive performance, with optimal durations ranging from 10–30 min (Brooks & Lack, 2006; Petit et al., 2018). Workplace-specific research reveals that strategic napping serves as a vital recovery mechanism for replenishing psychological and physiological resources depleted by work demands (Barnes, 2012). Longitudinal studies indicate that napping benefits follow diminishing returns patterns, with moderate-length naps showing optimal effects across multiple outcome domains (J. Liu et al., 2019). However, research also reveals potential negative consequences of suboptimal napping behaviors, with studies indicating that naps exceeding 30 min are associated with increased sleep inertia, temporary cognitive impairment, and heightened cardiovascular risks (L. Wang et al., 2022; Tassi & Muzet, 2000). These findings suggest that the relationship between napping and workplace outcomes is more complex than previously assumed, requiring careful consideration of duration parameters rather than treating napping as a uniformly beneficial behavior.

The theoretical and empirical evidence reviewed above converges on several key conclusions that extend the WNSF framework and provide specific guidance for understanding napping–satisfaction relationships. First, napping influences job satisfaction through multiple interconnected mechanisms involving resource recovery, cognitive enhancement, and emotional regulation that operate simultaneously rather than independently. Second, these mechanisms operate within optimal boundaries determined by sleep physiology and organizational time constraints, creating threshold effects that determine whether napping enhances or impairs workplace outcomes. Third, the relationship between nap duration and job satisfaction follows an inverted U-shaped pattern, reflecting the transition from beneficial to potentially counterproductive effects as duration increases beyond optimal levels. This integrated framework extends the WNSF by specifying precise boundary conditions under which sleep behaviors optimize workplace outcomes, moving beyond general assertions about sleep’s importance to identify specific parameters for maximizing benefits. The framework also reconciles apparently contradictory findings in the literature by recognizing that napping effects depend critically on duration parameters rather than mere occurrence, providing a theoretical foundation for understanding when and why napping enhances job satisfaction.

Integrating these findings with the perspectives of the WNSF, we can infer a curvilinear relationship between napping habits and job satisfaction. Based on the aforementioned analysis, this study proposes the following hypotheses regarding the relationship between napping habits and job satisfaction:

**Hypothesis** **1a:** *Compared to the non-napping group, the napping group will exhibit higher job satisfaction.*

**Hypothesis** **1b:** *The relationship between nap duration and job satisfaction is inverted U-shaped, implying the existence of an optimal point where nap duration maximizes its positive impact on job satisfaction.*

## 3. Research Design and Model Selection

### 3.1. Model Specification

Under specific conditions, the β estimates in the fixed effects model may be highly dependent on the sample—that is, overly sensitive to random errors in the given data. Suppose there are few within-group observations, or the change in x relative to y is small. Then, the within-group effect estimate of x on y might exhibit substantial bias relative to the true effect due to random factors. A drawback of the fixed effects model is the need to estimate the coefficients for each group indicator variable. This significantly reduces the model’s efficiency and increases the standard errors of the coefficient estimates. The problem becomes more severe when the within-group sample size is small, as the group effects alone can explain a large portion of the variation in the dependent variable. The random effects model partially pools information across groups through the between-group component, rendering the β estimates less variable (Gelman & Hill, 2007). Random effects estimates form a compromise between fixed effects models and mixed models, shrinking the α_j for deviating groups towards the average μ_α. This moves the β estimates away from the unstable fixed effects estimates and towards more stable (although possibly biased) estimates (Clark & Linzer, 2015). Given that our sample data have relatively few within-group samples, a random effects model is more suitable.

Certainly, scholars have suggested utilizing the Hausman (1978) specification test to examine whether the assumption of the random effects model, which presupposes the orthogonality of explanatory variables and group effects, has been violated. When the test results are significant, it indicates a correlation between x and α_j, implying that the random effects model should be abandoned in favor of the fixed effects model. However, in most applications, the true correlation between covariates and group effects is not entirely zero. Therefore, if the Hausman test cannot reject the null hypothesis of orthogonality, it is likely not because the true correlation is zero. Conversely, the test probably lacks sufficient statistical power to reliably distinguish between small correlations and zero correlation; when using the random effects model, even if the Hausman test does not find significant results, there will still be bias in the β estimates; of course, in many cases, if the biased (random effects) estimator provides sufficient variance shrinkage compared to the unbiased (fixed effects) estimator, the biased estimator might be preferable (Clark & Linzer, 2015). Thus, we compared the variance shrinkage of the fixed effects model and the random effects model and found that for most variables, the standard errors of the random effects model are smaller than those of the fixed effects model. This implies that the random effects model provides greater variance shrinkage for these estimates.

Therefore, to investigate whether napping and nap duration affect job satisfaction, the model designed in this study is as follows:

(1) $$Job\_Satisfaction_{it}=\alpha +\beta Nap_{it}+\gamma X_{it}+\mu_{i}+\varepsilon_{it}\;$$

(2) $$Job\_Satisfaction_{it}=\alpha +\beta_{1}Nap\_num_{it}+\beta_{2}Nap\_nu{m_{it}}^{2}+\gamma X_{it}+\mu_{i}+\varepsilon_{it}\;$$

Wherein, $Job\_Satisfaction_{it}$ represents the job satisfaction of individual i at time t. The core explanatory variable $Nap_{it}$ indicates whether individual i takes a nap at time t, and $Nap\_num_{it}$ represents the nap duration of individual i at time t. $Nap\_nu{m_{it}}^{2}$ is introduced to capture the curvilinear effect. $X_{it}$ is the control variable for individual i at time t. $\mu_{i}$ is the individual random effect, and $\varepsilon_{it}$ is the unobservable residual term.

### 3.2. Sample Selection and Data Source

The independent variables, dependent variables, and data utilized in this study originate from the China Family Panel Studies (CFPS) database. CFPS, an open-access database, allows data acquisition through registration and application. Embarking in 2010 and conducting surveys biennially (with the exception of 2011), CFPS represents a large-scale, longitudinal study, comprising a nationally representative sample of Chinese adults and children, obtained through random sampling. Surveys from 2016 to 2020 encompass a set of measurements related to satisfaction, including inquiries about the work environment, income, safety, time, promotions, and overall job satisfaction, while comprehensive survey items regarding job satisfaction are absent in other survey years. Notably, questions about individual-level napping behaviors, specifically the occurrence and duration of naps, are present in all survey years from 2010 to 2020, excluding 2012. Furthermore, this research contemplates the issue of endogeneity within the model, selecting sunlight duration as an instrumental variable. The data for sunlight duration is retrieved from the Wind Database, which encompasses daily, weekly, monthly, quarterly, semi-annual, and annual data for over 3000 regions in China, including provinces, cities, and counties.

This study also engaged in meticulous data-cleaning processes to ensure the derivation of reliable results. Initially, we selected sample data that was consistently reported from 2016 to 2020, identified via the unique individual identifier “pid”. Furthermore, we refined the samples based on two variables from the questionnaire: “current employment status” (variable name in the data: employ) and “currently attending school” (variable name in the data: qc1), thereby isolating samples of individuals who were employed and not attending school during the period from 2016 to 2020. Upon examining the obtained samples, we found that independent variable 1 (to nap or not) did not exhibit missing values. For handling missing values, we adopted the following approach: if independent variable 2 (nap duration) was greater than 0, it indicated napping, ensuring a logical consistency between independent variables 1 and 2. The majority of the control variables did not exceed 1.5% of their own quantities in missing values. We did not initially impute missing values for control variables; first, we removed samples with missing dependent variables (while retaining samples with “pid” consistently reported over three years). The dependent variable, job satisfaction, had 568 samples with missing values (constituting 4.64% of the data), which we removed, further retaining samples consistently reported over three years. Consequently, the proportion of missing values in control variables also decreased. In this study, the primary control variables included residence (urban or rural), age, gender, marital status, type of occupation, and highest educational attainment. While we attempted to incorporate sleep duration on workdays and income into the control variables, their excessive missing values (47.86% for income and 23.07% for sleep duration on workdays) precluded their inclusion in the analysis. However, we will explore these in subsequent sensitivity analyses.

The control variables we selected can have their missing values reasonably imputed from the values of the previous or following year. Naturally, for data across three periods within the control variables that are entirely absent (the missing values corresponding to “pid” are absent throughout the three years), we directly eliminate them. After operating in this manner, our independent variable 2 (nap duration) still had a 15.18% missing rate. We did not directly impute the missing values, but we eliminated all missing values of the core variables and ensured that the remaining data were reported for all three years (to ensure balanced panel data). We then examined the impact of the core independent variables on the dependent variable under the condition of having control variables. The results showed that the independent variables still have a significant impact on the dependent variable after such operations. To utilize the existing data for baseline regression analysis to the greatest extent, we performed imputation on the data without deleting the missing values of nap duration. In fact, whether to impute or not does not have a significant impact because we will match and introduce instrumental variables subsequently. After matching and introducing instrumental variables (since we have high requirements for the instrumental variables, any missing values in any year are unacceptable, i.e., our strict requirement is that the instrumental variable data for the individual must be complete for all three years), we found that the missing data for nap duration, imputed via model prediction method, were also basically deleted. Ultimately, in our baseline regression, we analyzed the data without matching and introducing instrumental variables (10,728 entries), while during the analysis of instrumental variable, sensitivity analysis, treatment effect model analysis, and PSM matching model, we used the data with matched and introduced instrumental variables for analysis (7401 entries). In this way, we can largely ensure the reliability and scientific nature of the model.

### 3.3. Variable Measurement

Independent variable 1: To nap or not. It is crucial to note that “noon break” typically refers to a period of rest or relaxation during midday, not necessarily involving sleep, whereas “nap” explicitly denotes a brief sleep during midday. These two may differ significantly in terms of time allocation and activity content, thereby potentially yielding different impacts and conclusions in research. The original questionnaire measured this by asking, “Do you currently have a habit of napping?” with 1 representing napping and 5 representing not napping. For ease of analysis, we subsequently use 0 to represent not napping. In the sample for our baseline regression, the proportion of samples not napping is 35.70% (N = 3830), while the percentage of those napping is 64.30% (N = 6898), totaling N = 10,728 samples.

Independent variable 2: Nap duration. The original questionnaire measured this with “How many minutes do you generally nap?” The unit of this variable is minutes, and for ease of interpretation, we will adjust its unit to hours in subsequent analyses. For samples without napping, we set the nap duration to 0. Nap duration ranges from 0 ≤ nap duration ≤ 240 min, that is, within a range of 0 to 4 h.

Dependent variable: Job satisfaction. The original questionnaire measured this with “Overall, how satisfied are you with this job?” (for studies using similar items, see Demerouti et al., 2012; Trougakos et al., 2008). Answer options range from 1 (very dissatisfied) to 5 (very satisfied). It is worth reiterating that the samples we retained are all in a working state (employed), and we did not include samples that are unemployed or job-waiting. Job satisfaction is the variable of our utmost concern, and we did not perform any imputation on it; we deleted any values with missing data, thus using the original data.

Control variables. In our research, there are a total of 6 available control variables, namely, residing in urban or rural areas (Replace with ‘Urban’ in the following), age, gender, marital status, occupation type, and highest educational level (Replace with ‘Education’ in the following). Past research indicates that age and educational level can impact job satisfaction (Lee & Wilbur, 1985), and of course, gender, marital status, and occupation type (Peng et al., 2022), as well as urban/rural and occupation type can also significantly impact job satisfaction (Friend & Burns, 1977). Therefore, we controlled for these in our research.

Detailed information about the variables in the research can be viewed through the descriptive statistics in Table 1.

### 3.4. Discussion on Endogeneity

This study primarily investigates the linear impact of napping (to nap or not) on job satisfaction and the curvilinear effect of nap duration on job satisfaction, recognizing that the aforementioned models may harbor endogeneity issues due to omitted variables. As previously mentioned, the substantial missing values for work income and weekday sleep duration prevent us from effectively incorporating them as control variables in the model for analysis. Both work income and weekday sleep duration can concurrently influence nap duration and job satisfaction. For instance, existing research indicates that higher performance rewards often correlate with elevated job satisfaction (Heywood & Wei, 2006), and higher-income groups tend to exhibit higher sleep efficiency and longer sleep duration (Sosso et al., 2021). The model may also omit unobservable variables (such as individual health status, lifestyle, and napping needs), which can simultaneously affect job satisfaction and the propensity to nap.

On the other hand, the model may also be subject to self-selection issues. Generally, nap duration is often related to organizational policies, working hours, and personal habits. Regardless of whether the organization has stringent regulations or individuals have good napping habits, the choice to nap and nap duration often vary. For example, in a relatively liberal organization, especially those with flexible working arrangements, whether employees choose to nap and the duration of naps are often at their discretion. Moreover, individuals who nap regularly may be more inclined to select work environments that permit napping, thereby attaining higher job satisfaction (Baxter & Kroll-Smith, 2005). This implies that, under certain circumstances, employees may choose whether to nap or determine the duration of naps based on organizational requirements and characteristics, or their own needs.

To tackle the endogeneity concerns stemming from reverse causation and omitted variables in our model, we utilize the duration of daylight in an individual’s residing city as an instrumental variable. It is well-documented that daylight exposure can extend continuous sleep duration. This is because reduced exposure to daylight and extended periods of darkness correlate with a prolonged biological night, attributed to the extended secretion of melatonin, leading to longer sleep spans (Stothard et al., 2017; Wehr, 1991; Wehr et al., 1993). Furthermore, the quality of sleep is intrinsically tied to daylight exposure. Pertinent studies have shown that exposure to white light, rich in short wavelengths during the day, correlates with enhanced evening fatigue and improved sleep quality (Viola et al., 2008), diminished sleep onset latency, and an increased accrual of slow-wave sleep (Wams et al., 2017). Notably, the timing of such light exposure appears to have a bearing on sleep, with findings suggesting that individuals exposed to >10 lx of light have more frequent nocturnal awakenings and reduced slow-wave sleep (Wams et al., 2017). The WNSF also postulates that macro-level variables, such as light exposure, have a profound impact on sleep quality (Crain et al., 2018). Digging deeper, evidence suggests that sleep deprivation augments negative emotions in the workplace the subsequent day (Zohar et al., 2005), and the choice to take a nap during the day, as well as the nap duration, is significantly influenced by the sleep quality and duration of the preceding night (C. Wang et al., 2019). Thus, it is reasonable to infer that the daylight duration of the previous day might influence an individual’s decision to nap and the duration of the nap on the following day. Yet, the duration of daylight remains orthogonal to the unobserved variables that influence job satisfaction, validating the scientific rationale behind using city daylight duration as an instrumental variable for napping. To further mitigate the concerns of omitted variables in our model, we have also incorporated a sensitivity analysis.

To alleviate the estimation bias induced by self-selection issues, this study employs both treatment effect models and propensity score matching (PSM) for estimation. The estimation principle of the two-step method in treatment effect models is similar to that of the two-stage least squares (2SLS), but it requires the endogenous variable to be a dummy variable. The first step of the two-step estimation involves utilizing the probit model to estimate the probability of an individual entering the napping group. The second step employs ordinary least squares (OLS) regression to obtain the coefficient estimation value for job satisfaction. PSM, proposed by Rosenbaum and Rubin (1983) to address the measurement issue of distance between individuals during matching, calculates the average treatment effect (ATE) through the following steps: ① Incorporate variables affecting both “to nap or not” and job satisfaction into the matching variables. ② Utilize the logit model to estimate the conditional probability of an individual entering the napping group, i.e., the propensity score, and test whether the matching results pass the balance test and whether the fit between the treatment group and the control group is optimized after matching. ③ Employ the propensity score values for kernel matching, calculating the average treatment effect on the treated (ATT) for “to nap or not”.

### 3.5. Validation of Control Variables Based on the LASSO Model

In pursuit of a more robust and reliable model, we scrutinized the efficacy of our chosen control variables through the LASSO model. Upon executing the LASSO regression and tuning the parameter lambda, we determined the variables that necessitated control. As can be discerned from Table 2, under the stipulation of lambda = 0.01, the control variable ‘age’ is excluded. Even when lambda is adjusted to 0.001, ‘age’ remains unincorporated. Consequently, in subsequent analyses, we did not include ‘age’ among the control variables.

### 3.6. Analytical Strategy and Methodological Approach

To ensure scientific rigor and causal validity in our inferences about the relationship between napping and job satisfaction, this study constructs a multi-layered, progressive analytical framework. This systematic strategy not only addresses various potential biases but also establishes a robust chain of evidence through cross-validation between methods.

First, baseline random effects regression models serve as the foundation of our analysis, establishing preliminary correlations between napping and job satisfaction by incorporating key control variables (including residential area, gender, marital status, education level, and occupation type). The choice of random effects models is based on sample characteristics and Hausman test results, effectively balancing the trade-off between estimation efficiency and bias. However, despite including multiple control variables, baseline models still face endogeneity challenges, particularly unobserved individual characteristics (such as health status and sleep preferences) and reverse causality that may lead to estimation bias. Therefore, relying solely on control variable methods cannot establish reliable causal relationships, prompting us to employ more advanced econometric methods.

Second, the application of instrumental variable (IV) methods is crucial for addressing endogeneity issues. The selection of sunlight duration as an instrumental variable is based on sufficient theoretical foundations and empirical testing, satisfying both relevance and exclusion conditions. This method isolates variations in napping induced by exogenous factors, thereby resolving reverse causality and omitted variable bias. We ensure the validity of instrumental variables through rigorous econometric tests (including F-statistics, Cragg–Donald Wald tests, and Kleibergen–Paap rk Wald tests). This approach is decisive in establishing the causal relationship between napping behavior and job satisfaction, particularly in verifying the existence of an inverted U-shaped relationship.

Third, sensitivity analysis is an indispensable component of our analytical framework, systematically assessing the sensitivity of results to unobserved confounding factors. By constructing a comparative benchmark (using occupation type as a reference), we can quantify how strong potential omitted variables would need to be to alter our research conclusions. This transparent methodological assessment is critical for verifying the robustness of causal inferences, especially considering that we cannot include certain potentially relevant variables (such as work income and workday sleep duration) in the basic model due to their high missing rates. Without this analysis, we would be unable to effectively evaluate the extent to which results are influenced by omitted variables.

Fourth, treatment effect models and propensity score matching (PSM) methods offer complementary methodological approaches to addressing self-selection issues. Treatment effect models explicitly model the napping decision process through two-stage estimation, while PSM creates matched samples based on observable characteristics to minimize selection bias. The combined application of these two methods is particularly important because they are based on different statistical principles and can cross-validate the consistency of results. The core advantage of PSM is that it does not rely on linear assumptions but directly compares individuals with similar characteristics, thus providing an important supplement to parametric models. If both methods show that napping has a significant impact on job satisfaction, the credibility of our inferences is greatly enhanced.

Finally, we employ multiple robustness testing strategies to systematically assess the stability of results. Quantile regression analysis allows us to test whether the effect of napping is consistent at different points in the job satisfaction distribution, revealing potential effect heterogeneity. Gender-based group analysis examines the applicability of findings across male and female populations, providing key information for understanding the universality of napping effects. Additionally, by expanding the set of control variables (including work income, nighttime sleep duration on workdays, and life stress), we can comprehensively assess the sensitivity of results to model specifications. In particular, the inclusion of these additional control variables compensates for factors omitted from the baseline model due to data limitations, further enhancing the reliability of causal inferences.

This hierarchical, multi-faceted analytical strategy is necessary because no single method can adequately address the complex challenges of causal inference. Various methods are based on different identifying assumptions and address different types of bias: IV methods handle endogeneity, PSM and treatment effect models resolve self-selection issues, sensitivity analysis evaluates the impact of unobserved heterogeneity, and multiple robustness tests verify that results do not depend on specific model specifications.

## 4. Empirical Results and Analysis

### 4.1. Baseline Regression

Table 3 reports the baseline regression results of napping behavior on job satisfaction. Column (1) represents only control variables being included, column (2) incorporates the independent variable “to nap or not”, and column (3) introduces the independent variables of “nap duration” and its squared term (Sq.Nap duration). After controlling for relevant variables, the “to nap or not” in column (2) is significant at the 0.1% level, indicating that napping indeed helps to enhance job satisfaction. Both “nap duration” and its squared term in column (3) are significant at the 0.1% level, suggesting a U-shaped effect of nap duration on job satisfaction.

### 4.2. Addressing Endogeneity Issues Arising from Reverse Causality and Omitted Variables: The Instrumental Variable Approach

The relationship between napping behavior and job satisfaction may be subject to reverse causality. Lower job satisfaction, indicative of heightened work stress and fatigue, might predispose individuals to opt for napping as a means to alleviate tiredness, thereby making napping behavior more prevalent. Additionally, omitted variables potentially influencing both napping and job satisfaction could introduce endogeneity bias. To mitigate the impact of endogeneity on the model, this study employs the average daily sunlight duration in an individual’s city as an instrumental variable for both the decision to nap and nap duration, conducting a two-stage least squares estimation. Table 4 reports the results of the instrumental variable tests. Taking column (1) in Table 4 as an example, both the Cragg–Donald Wald F statistic and the Kleibergen–Paap rk Wald F statistic exceed the critical value at the 10% level; furthermore, with an F-value of 33.39 in the two-stage estimation results, and considering that the critical value at a 10% bias level is 16.38 (Stock et al., 2002), weak instrumental variable issues are not present. The instrumental variable estimation results show that the coefficient for the decision to nap is 1.1964 and is significant at the 0.1% level, indicating that the group that naps has higher job satisfaction compared to the non-napping group. Column (2) examines the causal relationship between the squared nap duration and job satisfaction, and the results uphold the significant inverted U-shaped impact of nap duration on job satisfaction. Column (3) explores the causal relationship between nap duration and job satisfaction, and the results remain robust and are significant at the 0.1% level (however, this is not our primary focus, as existing research has already demonstrated that excessive nap duration can have various negative effects). In summary, napping is a crucial factor in enhancing satisfaction, and there exists a curvilinear effect of napping on job satisfaction. Appropriate nap durations are beneficial, while excessive napping can yield negative outcomes for job satisfaction.

### 4.3. Addressing Endogeneity Issues Arising from Omitted Variables: A Sensitivity Analysis

**Figure 1:**
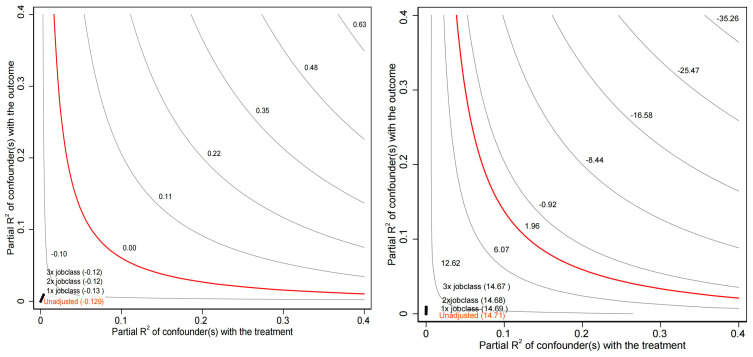
Contour plots of coefficient β for ‘nap duration’ (**left**) and corresponding T-statistic (**right**).

**Figure 2:**
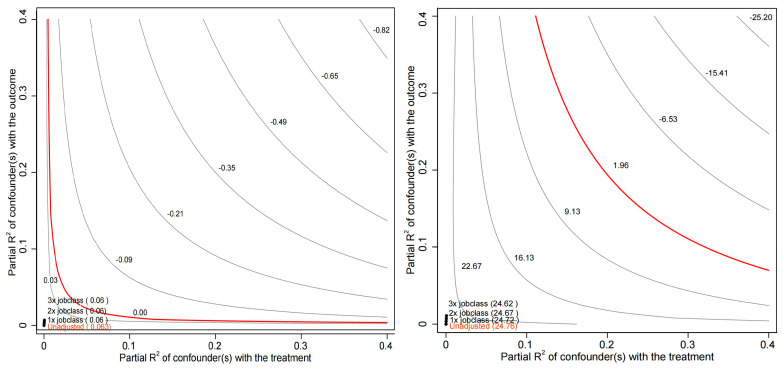
Contour plots of coefficient β for ‘to nap or not’ (**left**) and corresponding T-statistic (**right**).

In this study, omitted variables are inevitable due to the impact of available data and missing values. As mentioned earlier, due to the excessive missing values of sleep duration on workdays and work income, we did not include them in the analysis, which resulted in an omitted variable issue. Furthermore, omitted variables, such as an individual’s health status and the distance between home and the workplace, may also interfere with our model. Complicating matters, these factors might interact with each other or produce other nonlinear impacts. Therefore, we employ a sensitivity analysis to demonstrate the robustness of the conclusions of this study (Cinelli et al., 2024). To better illustrate the robustness of the model, we introduce a comparison variable—occupational type, as we believe that the type of occupation is a crucial factor affecting individual napping behavior and job satisfaction. The sensitivity analysis results for the inverted U-shaped impact of nap duration on job satisfaction can be seen in Figure 1, while the sensitivity analysis results for the impact of ‘to nap or not’ on job satisfaction can be observed in Figure 2.

In Figure 1, the contour on the left represents regression coefficient values β, with the red line indicating β = 0. The four numerical points (located at the bottom left of the graph) respectively represent scenarios: without incorporating omitted variables, introducing omitted variables of the same strength as occupational type, introducing omitted variables with twice the strength of occupational type, and introducing omitted variables with three times the strength of occupational type. The coefficient valuesβfor the corresponding econometric models are reported in parentheses at the four numerical points and are all observed to be greater than zero, meaning that all four numerical points are situated to the left of the red line. This implies that even when introducing omitted variables with three times the strength of occupational type, the original estimated coefficient will not change from positive to negative. The contour on the right of Figure 1 represents the T-statistic, with the red line indicating 1.96 (the critical value for a 95% confidence interval). The four numerical points, consistent with the previous figure, respectively indicate scenarios of introducing omitted variables with 0 to 3 times the strength of occupational type. The T-statistics for the corresponding econometric models are reported in parentheses at the four numerical points. The T-statistics for the three numerical points, which introduce omitted variables with 0 to 3 times the strength of occupational type, are less than 1.96 and are located to the left of the red line. This implies that even when introducing omitted variables with three times the strength of occupational type, the original estimated coefficient will not change from significant to non-significant. Therefore, the inverted U-shaped impact of nap duration on job satisfaction still holds even when considering high-strength omitted variables. The conclusions in Figure 2 are consistent, and the interpretation process is the same, so we will not elaborate further.

### 4.4. Addressing Endogeneity Issues Caused by Self-Selection: Treatment Effect Model

In addition to the endogeneity issues caused by reverse causality and omitted variables, the model may also be subject to significant self-selection issues. This study employs the treatment effect model to mitigate estimation biases caused by self-selection problems. Since the endogenous variable, “to nap or not”, is a binary dummy variable, it is suitable for the treatment effect model. Table 5 reports the estimation results of the two-step method of the treatment effect model. The estimation results in column (1) show that the coefficient of “to nap or not” is significant at the 5% level in the first-stage Probit regression, indicating a significant positive correlation between city sunlight duration and whether to nap. Since the two-step method cannot test whether the model has endogeneity issues, this study uses maximum likelihood estimation to obtain endogeneity test results. The Wald test for endogeneity rejects the null hypothesis that the model does not have endogeneity issues at the 0.1% significance level, indicating that whether to nap does have endogeneity issues. The results of the treatment effect model show that the impact coefficient of whether to nap is 2.428 and is significant at the 5% level.

### 4.5. Addressing Endogeneity Issues Arising from Self-Selection: Propensity Score Matching

Propensity score matching (PSM) can also mitigate, to a certain extent, the estimation biases brought about by self-selection issues. In this study, we achieve the best-fitting effect of covariates in the first and second forms by comparing the maximum likelihood values of different models (Imbens, 2015). Calculations are performed for whether residing in urban or rural areas, gender, age, educational level, marital status, type of work, and parallel items of educational level, and a Logit regression is conducted to estimate the propensity scores. Table 6 reports the results of kernel matching. The kernel matching results, with overall job satisfaction as the outcome variable, show that the Average Treatment Effect of labor mobility is 21.36%, significant at the 0.1% level. When the outcome variable is changed to satisfaction with the working environment, the results remain robust.

**Figure 3:**
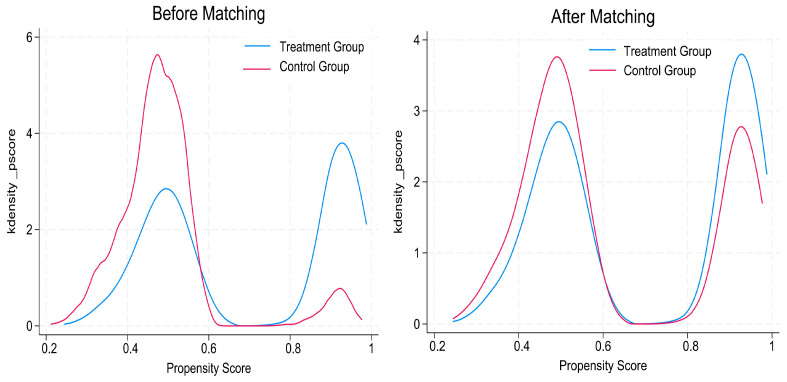
Fitted propensity score values after matching.

As shown in Figure 3, the degree of fit between the treatment group and the control group after matching is superior to that before matching.

## 5. Robustness Check

### 5.1. Quantile Regression

Quantile regression, aiming to minimize the weighted average of the absolute values of residuals, is less susceptible to outliers compared to OLS estimates and can more comprehensively identify the impact of explanatory variables on the dependent variable at different quantiles. As analyzed earlier, napping significantly enhances job satisfaction, and nap duration has a notable inverted U-shaped effect on job satisfaction. However, the job satisfaction derived from napping might exhibit heterogeneity among individuals in different industries. Freelancers might experience greater uncertainty regarding whether to nap and the duration of naps. Furthermore, a longer nap might imply a shorter working time, which could also interfere with an individual’s job satisfaction. This study examines the impact of napping and Nap Duration on the 0.2, 0.4, and 0.8 quantiles of job satisfaction. The results in Table 7 indicate that at each quantile, both napping and nap duration exert a significant positive impact on job satisfaction. The outcomes from the quantile regression suggest that the group who naps has higher job satisfaction compared to the non-napping group. Moreover, an inverted U-shaped effect of nap duration on job satisfaction is observed, implying that an appropriate nap duration can enhance an individual’s job satisfaction, while excessively long or short naps may not be optimal choices.

### 5.2. Gender-Based Group Regression

In the realm of napping, a study discovered that within the group who naps four or more days per week, men constitute 21.2%, while women make up 17.1%. Moreover, napping behaviors are complex and may be associated with various antecedents and consequences across different subpopulations (Furihata et al., 2016). Gender disparities might also be present in job satisfaction. For instance, men tend to be more satisfied than women with aspects like working hours, career advancement opportunities, and workload, whereas women exhibit greater satisfaction than men in areas such as relationships with colleagues and contributions to society (Kifle & Hailemariam Desta, 2012). Consequently, we also explore the gender-specific differences in the impact of napping behaviors on job satisfaction. The results, presented in Table 8, reveal that at a 0.1% significance level, both men and women who nap exhibit higher job satisfaction than those who do not, and the curvilinear effect holds true for both genders, indicating that optimal job satisfaction is achieved with an appropriate nap duration.

### 5.3. Substituting the Dependent Variable and Adding Control Variables

In this section, we replace the overall job satisfaction variable with job environment satisfaction in the regression analysis. Additionally, we incorporate three new control variables: work income, nighttime sleep duration on working days, and life stress. As shown in Table 9, the results indicate that the decision to nap continues to have a significantly positive effect on both job environment satisfaction and overall job satisfaction. Furthermore, nap duration still exhibits a significant inverted U-shaped relationship with both outcome variables. These findings are consistent with the baseline regression results using overall job satisfaction, suggesting that the model in this study demonstrates solid robustness.

## 6. Results and Discussion

Our empirical analysis reveals that napping significantly enhances job satisfaction, and nap duration exhibits an inverted U-shaped relationship with job satisfaction, with optimal benefits occurring within the 20–30 min range. These findings have withstood rigorous methodological scrutiny through instrumental variable analysis, treatment effect models, sensitivity analysis, and propensity score matching, while demonstrating consistency across quantile regression, gender-differentiated analysis, and alternative outcome measures.

### 6.1. Theoretical Contributions

Our research makes three fundamental theoretical contributions that advance sleep and organizational psychology research while providing compelling empirical validation for several theoretical frameworks. First, we provide the first systematic empirical validation of the inverted U-shaped relationship between nap duration and job satisfaction, transforming a theoretically plausible proposition into empirically demonstrated reality. The observed inverted U-shaped relationship provides compelling empirical validation for resource conservation theory (Hobfoll, 1989), demonstrating that employees face optimization challenges in allocating finite time and energy resources between work activities and recovery behaviors. The initial positive slope reflects the resource replenishment benefits of strategic napping, where brief sleep episodes effectively restore depleted cognitive and emotional resources without substantial time costs, while the declining portion beyond 30 min reveals how extended napping creates temporal conflicts with work responsibilities and generates stress that offsets recovery benefits.

Our identification of optimal nap duration within the 20–30 min range provides empirical support for sleep stage theories and aligns with previous physiological research by Brooks and Lack (2006), who demonstrated that naps of this duration allow individuals to experience restorative benefits of stage 1 and 2 sleep while avoiding deeper slow-wave sleep that leads to sleep inertia. This finding refines WNSF’s fundamental assumption that sleep quality and quantity uniformly impact energetic activation (Crain et al., 2018). Instead, we reveal a non-linear pattern where optimal, rather than maximum, sleep duration yields superior outcomes, aligning with resource conservation theory principles. This reconciles contradictory literature where some studies reported positive effects of napping (Hayashi et al., 2004) while others found negative outcomes from excessive napping (Mullins et al., 2014), demonstrating that napping effects depend critically on duration parameters rather than mere occurrence.

The declining portion of the inverted U-curve beyond 30 min illuminates multiple theoretical mechanisms that transform beneficial recovery behaviors into counterproductive activities. Sleep inertia theory explains the physiological basis for diminished satisfaction following extended napping, as longer sleep episodes increase the probability of entering deep sleep stages that require substantial recovery time upon awakening (Hilditch et al., 2016). Simultaneously, social cognitive theory provides explanatory power for understanding why extended napping reduces job satisfaction through workplace evaluation processes, as employees engaging in prolonged workplace napping may experience cognitive dissonance regarding their organizational commitment, supporting previous research by Mullins et al. (2014). Furthermore, perceived social evaluation risks from colleagues and supervisors create additional psychological costs that accumulate as nap duration increases beyond socially acceptable thresholds, as documented by Takahashi et al. (2004).

The gender consistency of our findings across male and female employees provides important theoretical insights regarding the universality of sleep-satisfaction relationships. Despite documented gender differences in sleep patterns and workplace experiences, both male and female employees demonstrate identical inverted U-shaped relationships between nap duration and job satisfaction. This consistency suggests that the underlying physiological and psychological mechanisms transcend gender-specific workplace dynamics, supporting the generalizability of our theoretical framework. As Centofanti et al. (2016) noted, individual differences may moderate the napping–satisfaction relationship, yet our findings demonstrate consistent optimal parameters across different subgroups, suggesting that physiological constraints operate universally while recognizing potential individual variations in implementation.

Second, we extend the work, non-work, and sleep framework (WNSF) by demonstrating how sleep behaviors operate within cultural contexts where napping is normalized, expanding the framework’s scope to include midday sleep episodes alongside nighttime sleep and making it more applicable to diverse sleep patterns in modern work environments. While the original framework by Crain et al. (2018) identified sleep as critical for work attitudes but did not fully articulate boundary conditions or precise mechanisms, our research demonstrates that sleep’s influence operates within optimal boundaries rather than following a simple “more is better” principle. This theoretical advancement enables more precise predictions about workplace interventions and provides a foundation for developing evidence-based organizational policies regarding employee recovery behaviors.

Third, our rigorous methodological approach, including instrumental variable techniques, sensitivity analyses, and matching models, strengthens causal inferences regarding the nap-satisfaction relationship and addresses critical limitations of previous correlational studies. By establishing causal relationships through quasi-natural experimental design with longitudinal data, we provide methodological advancement that transforms theoretical propositions about sleep-work relationships into empirically validated findings with clear policy implications.

### 6.2. Practical Implications

Our findings provide direct guidance for organizational napping policy development based on empirical evidence. Organizations should implement napping protocols specifying 20–30 min duration limits, aligning with Brooks and Lack’s (2006) physiological research demonstrating optimal recovery benefits within this timeframe. Dedicated napping spaces should include appropriate environmental conditions such as dim lighting, low noise levels, and comfortable resting arrangements (Anthony & Anthony, 2005; Takahashi, 2003). Strategic scheduling during post-lunch circadian dips (1:00–3:00 PM) maximizes napping benefits, as confirmed by circadian rhythm research (Monk, 2005; Campbell et al., 2005). Several organizations have successfully implemented such policies, with Japanese companies demonstrating enhanced employee productivity and satisfaction through institutionalized napping practices (Steger, 2006; Takahashi et al., 2004).

However, implementation must account for individual differences and job constraints, offering alternative recovery activities for employees unable to nap during work hours. Organizations should also implement evaluation mechanisms to assess policy effectiveness and refine protocols based on employee outcomes while being mindful of potential health implications associated with excessive napping duration (Ju & Choi, 2013).

### 6.3. Limitations and Future Research Directions

Our study’s primary limitations include cultural specificity (Chinese sample), exclusive focus on job satisfaction outcomes, and inability to assess nap quality or timing precision. The cultural context limitation is particularly important given that workplace napping acceptance varies significantly across cultures (Cheung et al., 2021). Future research should examine cross-cultural generalizability and extend the inverted U-shaped relationship to behavioral outcomes such as performance and creativity. Individual difference moderators such as chronotype and sleep preferences warrant investigation, as research by Duggan et al. (2018) and Arora et al. (2006) suggests substantial variability in physiological responses to daytime napping. Additionally, as emphasized by Driskell and Mullen (2005), future studies should consider nap timing and post-nap intervals alongside duration parameters. Longitudinal studies with granular sleep tracking could provide more nuanced insights into how napping pattern consistency influences workplace outcomes.

## Figures and Tables

**Table 1:** Descriptive statistics.

Variable	Mean	SD	Min	Max	1	2	3	4	5	6	7	8	9	10	11	12
Job satisfaction (1)	3.497	0.873	1	5	1											
Nap duration (2)	0.599	0.64	0	4	0.160 ***	1										
To nap or not (3)	0.643	0.479	0	1	0.057 ***	0.696 ***	1									
Urban (4)	0.555	0.497	0	1	0.067 ***	−0.023 *	0.030 **	1								
Gender (5)	0.579	0.494	0	1	−0.033 ***	−0.007	−0.034 ***	−0.01	1							
Age (6)	33.264	5.864	16	44	0.000	0.061 ***	0.145 ***	−0.029 **	−0.055 ***	1						
Education (7)	4.201	1.714	0	10	0.131 ***	0.064 ***	0.184 ***	0.284 ***	−0.042 ***	−0.041 ***	1					
Marital Status (8)	0.824	0.381	0	1	−0.01	0.023 *	0.026 **	−0.057 ***	−0.124 ***	0.419 ***	−0.035 ***	1				
Occupation Type_dum1 (9)	0.219	0.414	0	1	−0.126 ***	0.035 ***	−0.029 **	−0.354 ***	−0.117 ***	0.175 ***	−0.302 ***	0.133 ***	1			
Occupation Type_dum2 (10)	0.149	0.356	0	1	0.056 ***	0.022 *	−0.016	0.032 ***	0.041 ***	0.045 ***	−0.049 ***	0.089 ***	−0.221 ***	1		
Occupation Type_dum3 (11)	0.004	0.065	0	1	−0.026 **	0.002	0.01	−0.019	0.007	0.027 **	−0.023 *	0.012	−0.035 ***	−0.027 **	1	
Occupation Type_dum4 (12)	0.606	0.489	0	1	0.079 ***	−0.041 ***	0.041 ***	0.288 ***	0.044 ***	−0.186 ***	0.317 ***	−0.181 ***	−0.657 ***	−0.518 ***	−0.082 ***	1
Occupation Type_dum5 (13)	0.022	0.147	0	1	−0.031 **	−0.016	−0.021 *	−0.031 **	0.082 ***	0.005	−0.077 ***	0.008	−0.080 ***	−0.063 ***	−0.01	−0.186 ***

Note: * *p* < 0.05, ** *p* < 0.01, *** *p* < 0.001.

**Table 2:** Control variables selected, based on the LASSO model.

LASSO	Lambda	Urban	Gender	Age	Education	Marital Status	Occupation Type	To Nap or Not	Sq.Nap Duration	Nap Duration
Job Satisfaction	0.14	No	No	No	No	No	No	No	No	Yes
	0.11	No	No	No	No	No	No	Yes	No	Yes
	……									
	0.01	Yes	Yes	No	Yes	Yes	Yes	Yes	Yes	Yes
	……									
	0.001	Yes	Yes	No	Yes	Yes	Yes	Yes	Yes	Yes

Note: Sq.Nap duration represents the square term of nap duration.

**Table 3:** The impact of napping on job satisfaction: baseline regression results.

	(1)	(2)	(3)
	Job Satisfaction	Job Satisfaction	Job Satisfaction
To nap or not		0.0699 ***	
		(0.0169)	
Sq.Nap duration			−0.1214 ***
			(0.0158)
Nap duration			0.4283 ***
			(0.0297)
Control variables	YES	YES	YES
*N*	10,728	10,728	10,728
R^2^	0.0293	0.0306	0.0602

Standard errors in parentheses, robust to heteroskedasticity.* *p* < 0.05, ** *p* < 0.01, *** *p* < 0.001. Likewise, in the following text, without further ado.

**Table 4:** Impact of napping on job satisfaction: instrumental variable method.

	(1)	(2)	(3)
	Job Satisfaction	Job Satisfaction	Job Satisfaction
To nap or not	1.1964 ***		
	(0.3381)		
Sq.Nap duration		−0.9737 ***	Controlled
		(0.2895)	
Nap duration		Controlled	1.6093 ***
			(0.3667)
Control variables	YES	YES	YES
N	7401	7401	7401
R^2^	0.0325	0.0210	0.0411
First-stage F-value	33.39	26.66	54.72
Cragg–Donald Wald F statistic	33.387	30.436	68.758
Kleibergen–Paap rk Wald F statistic	52.525	26.662	54.719
*p*-value	0.000	0.000	0.000

**Table 5:** Impact of ‘to nap or not’ on job satisfaction: treatment effect model.

	(1)	(2)
	To nap or not	Job satisfaction
Duration of illumination	0.023 *	
	(0.012)	
To nap or not		2.428 *
		(1.033)
Control variables	YES	YES
N	7401	7401
Wald test Chi^2^	—	150.70
*p*-value	0.000	0.000
Pseudo R^2^	0.0224	—

**Table 6:** Impact of ‘to nap or not’ on job satisfaction: Kernel matching.

	Outcome Variables	Treated	Controls	ATT	Standard Error	t-Value
Kernel Matching	Overall job satisfaction	3.7437	1.8791	1.8645	0.0319	58.44
	Job environment satisfaction	3.7002	2.6100	1.0902	0.8436	12.92

**Table 7:** The impact of napping on job satisfaction: quantile regression.

	Q20	Q20	Q40	Q40	Q80	Q80
	Job Satisfaction	Job Satisfaction	Job Satisfaction	Job Satisfaction	Job Satisfaction	Job Satisfaction
To nap or not	0.0677 *		0.0780 ***		0.1044 ***	
	(0.0274)		(0.0196)		(0.0225)	
Sq.Nap duration		−0.1255 ***		−0.1161 ***		−0.0962 ***
		(0.0304)		(0.0204)		(0.0252)
Nap duration		0.4626 ***		0.4273 ***		0.3522 ***
		(0.0555)		(0.0373)		(0.0460)
Control variables	YES	YES	YES	YES	YES	YES
*N*	10,728	10,728	10,728	10,728	10,728	10,728

**Table 8:** The impact of napping on job satisfaction: gender differences.

	Male	Female	Male	Female
	Job Satisfaction	Job Satisfaction	Job Satisfaction	Job Satisfaction
To nap or not	0.0738 ***	0.0864 ***		
	(0.0215)	(0.0262)		
Sq.Nap duration			−0.1276 ***	−0.1153 ***
			(0.0189)	(0.0282)
Nap duration			0.4398 ***	0.4224 ***
			(0.0370)	(0.0502)
Control variables	YES	YES	YES	YES
*N*	6210	4518	6210	4518
R^2^	0.0267	0.0270	0.0578	0.0540

**Table 9:** Substituting the dependent variable and adding control variables.

	(1)	(2)	(3)	(4)
	Job Environment Satisfaction	Job Environment Satisfaction	Job Satisfaction	Job Satisfaction
To nap or not	0.0675 ***		0.0662 *	
	(0.0185)		(0.0309)	
Sq.Nap duration		−0.0630 ***		−0.0903 ***
		(0.0166)		(0.0281)
Nap duration		0.2225 ***		0.2767 ***
		(0.0325)		(0.0562)
Control variables	YES	YES	YES	YES
Add new control variables				
Income			0.0893 ***	0.0873 ***
			(0.0191)	(0.0192)
Nighttime sleep duration on working days			0.0349 *	0.0318 *
			(0.0514)	(0.0513)
Life stress			−0.0759	−0.0753
			(0.0429)	(0.0424)
*N*	10,728	10,728	2746	2746
R^2^	0.0197	0.0235	0.0408	0.0603

## Data Availability

The data for this study originate from the China Family Panel Studies (CFPS), available in both Chinese and English. CFPS provides comprehensive user guidelines and resources. Access to the CFPS data requires an application, as direct sharing or redistribution by individuals is not permitted. Detailed application information and video instructions are available upon account registration at http://www.isss.pku.edu.cn/cfps/download/login (accessed on 18 June 2023). Informed consent was obtained from all subjects involved in the study, as the CFPS database has undergone its own institutional ethical review.
